# Transitional States in Ligand-Dependent Transformation of the Aryl Hydrocarbon Receptor into Its DNA-Binding Form

**DOI:** 10.3390/ijms21072474

**Published:** 2020-04-02

**Authors:** Anatoly A. Soshilov, Stefano Motta, Laura Bonati, Michael S. Denison

**Affiliations:** 1 Department of Environmental Toxicology, University of California, Davis, CA 95616, USA; soshilov@gmail.com; 2 Department of Earth and Environmental Sciences, University of Milano-Bicocca, 20126 Milan, Italy; stefano.motta@unimib.it (S.M.); laura.bonati@unimib.it (L.B.)

**Keywords:** aryl hydrocarbon receptor, AhR, heat shock protein 90, hsp90, 2,3,7,8-tetrachlorodibenzo-p-dioxin, TCDD, aryl hydrocarbon receptor nuclear translocator, ARNT, molybdate

## Abstract

The aryl hydrocarbon receptor (AhR) is a ligand-activated transcription factor that mediates the biological and toxicological effects of an AhR lacking the entire PASB structurally diverse chemicals, including halogenated aromatic hydrocarbons. Ligand-dependent transformation of the AhR into its DNA binding form involves a ligand-dependent conformational change, heat shock protein 90 (hsp90), dissociation from the AhR complex and AhR dimerization with the AhR nuclear translocator (ARNT) protein. The mechanism of AhR transformation was examined using mutational approaches and stabilization of the AhR:hsp90 complex with sodium molybdate. Insertion of a single mutation (F281A) in the hsp90-binding region of the AhR resulted in its constitutive (ligand-independent) transformation/DNA binding in vitro. Mutations of AhR residues within the Arg-Cys-rich region (R212A, R217A, R219A) and Asp371 (D371A) impaired AhR transformation without a significant effect on ligand binding. Stabilization of AhR:hsp90 binding with sodium molybdate decreased transformation/DNA binding of the wild type AhR but had no effect on constitutively active AhR mutants. Interestingly, transformation of the AhR in the presence of molybdate allowed detection of an intermediate transformation ternary complex containing hsp90, AhR, and ARNT. These results are consistent with a stepwise transformation mechanism in which binding of ARNT to the liganded AhR:hsp90 complex results in a progressive displacement of hsp90 and conversion of the AhR into its high affinity DNA binding form. The available molecular insights into the signaling mechanism of other Per-ARNT-Sim (PAS) domains and structural information on hsp90 association with other client proteins are consistent with the proposed transformation mechanism of the AhR.

## 1. Introduction

The aryl hydrocarbon receptor (AhR) is a ligand-dependent transcription factor that mediates the biological and toxic effects of 2,3,7,8-tetrachlorodibenzo-p-dioxin (TCDD, dioxin) and a structurally diverse range of chemicals [1,2]. In normal physiology, the AhR appears to be important in development; it plays a key regulatory role in immune responses and disease [1,3,4,5,6]. Numerous endogenous ligands of the AhR have been proposed, and while none have been established with certainty, tryptophan and indole metabolites are likely candidates [1,2,7,8]. Exposure to dioxin-like compounds results in adverse effects that are mediated by AhR-dependent transcriptional activation [1,9,10]. However, while AhR-dependent toxic effects are highly species-specific, differences in ligand binding affinity among species do not fully account for the observed variations [11]. It has been previously hypothesized that the species differences in biochemical properties of the AhR complex may somehow contribute to the differences in AhR-dependent toxic outcomes, emphasizing the need for better understanding of the process of ligand-dependent activation of the AhR.

In the absence of ligand, the AhR is present in a cytosolic complex with several proteins, including hsp90, which protects the AhR from degradation, helps to anchor the inactive complex within the cytoplasm, and maintains the AhR in an inactive state by blocking its nuclear localization sequence and AhR nuclear translocator (ARNT) dimerization interfaces [1,12,13,14,15,16]. Ligand binding results in nuclear translocation of the AhR protein complex and simultaneously leads to conversion of the AhR into its high affinity DNA binding form in a process termed AhR transformation [14,15,17,18]. At some point in this process, the AhR dissociates from the associated chaperone proteins and dimerizes with the homologous nuclear protein ARNT [15,17,18,19]. The AhR appears to remain associated with hsp90 through nuclear translocation, and hsp90 may be directly displaced by ARNT in the process that requires the AhR PASA (Per-ARNT-Sim A) dimerization domain [19,20,21,22]. While ligand binding may directly contribute to the destabilization of hsp90 bound within the AhR PASB (Per-ARNT-Sim B) ligand binding domain (LBD), located C-terminal to the PASA domain, it does not fully displace hsp90 from the AhR [16,21,23]. Hsp90 binding to the liganded AhR presumably results from its interaction with a second hsp90 binding site located within the AhR DNA-binding bHLH (basic Helix-Loop-Helix) domain and, possibly, lower affinity interactions with the PASB domain [16,21,24]. While these reports, taken together, suggest a multi-step mechanism of ligand-dependent AhR transformation, direct experimental evidence of such mechanism is lacking. Molecular insights into the signaling mechanism of the PAS domain are mostly documented in other proteins [25]. In both the Photoactive Yellow Protein (PYP) and the plant phototropins, light induces a conformational change within the PAS domain that mainly involves the β-sheet surface and propagates to the effector domain [26,27]. The oxygen sensor protein FixL also undergoes a conformational change in the β-sheet of the PAS domain, following ligand binding to the heme group [28]. Finally, citrate binding to the PAS domain of sensor histidine kinase CitA was shown to induce a flexion of the central β-sheet around the ligand that then propagates to the transmembrane domain of the protein [29]. Although the three-dimensional structure of the AhR PASB domain has been obtained by homology modeling [30], and both ligand binding [31] and dimerization with ARNT [32,33] have been computationally simulated, to date, the molecular effects of ligand binding on the mechanism of AhR transformation are not yet well understood.

The complexity of this mechanism, which consists of ligand binding, a ligand-dependent conformational change(s), hsp90 dissociation/displacement, and dimerization with ARNT, contributes to the experimental difficulty in examining the distinct steps of this mechanism. Characterization of presumed transitional states requires functional examination of the AhR during instances of incomplete AhR transformation, specifically when only a fraction of ligand-bound AhR is fully transformed into its high affinity DNA binding-competent AhR:ARNT dimer. Incomplete AhR transformation would be manifested as decreased transformation efficiency, defined as a ratio of DNA-bound to ligand (TCDD)-bound AhR forms. In fact, dramatic species differences in transformation efficiency have been previously reported, with human and mouse AhRs displaying low levels of transformation efficiency in comparison with other studied species [34,35]. These results, combined with identification of nuclear AhR:hsp90 complexes [19,20,22], suggest that incomplete AhR transformation and, therefore, intermediate transitional states of transformed AhR complex may indeed exist and could be experimentally exploited. Accordingly, here, we describe the results of studies using a combination of mutagenesis, immunoprecipitation, and molybdate stabilization of hsp90:protein complexes to investigate transitional states in the mechanism of AhR transformation into its high affinity DNA binding form. 

## 2. Results

The initial observation that led to the study of the mechanism of AhR transformation was the previously reported dramatic difference in DNA binding activity between in vitro synthesized ligand-activated wild type(wt)AhR and AhRdPASB, an AhR lacking the entire PASB ligand binding domain and active in the absence of ligand (i.e., constitutively active) [21]. While both of these AhR proteins were expressed at similar levels, the constitutively active AhRdPASB demonstrated a two-fold higher level of transformation/DNA binding compared to the ligand-activated wtAhR. This result suggested that the wtAhR was not maximally transformed by ligand in this system, and indeed, when calculated, its transformation efficiency (measured as a ratio of molar amount of [^3^H]TCDD bound-AhR to [^32^P]dioxin-responsive element (DRE)-bound AhR) was only about 50% [21], indicating the existence of inhibitory factors/mechanisms. Therefore, mutagenesis analysis could be utilized to further study the underlying mechanisms and, specifically, to identify AhR mutations that would alter AhR transformation efficiency.

### 2.1. Study of AhR Transformation through Mutagenesis within the PASB Domain

The ligand-binding PASB domain harbors one of the hsp90-binding sites in the AhR, and it exerts an inhibitory effect on AhR activation [16,21,24,36,37,38,39]. Given its key role in initiation of AhR transformation [21], it is conceivable that the PASB domain contains amino acid residues critical to this process. Accordingly, mutagenesis experiments were primarily focused on this domain. In particular, given that in most PAS domains signals propagate to and through the central β-sheet [25,26,27,28,29], mutagenesis was performed on residues spanning this region of the PASB domain. 

**Figure 1:**
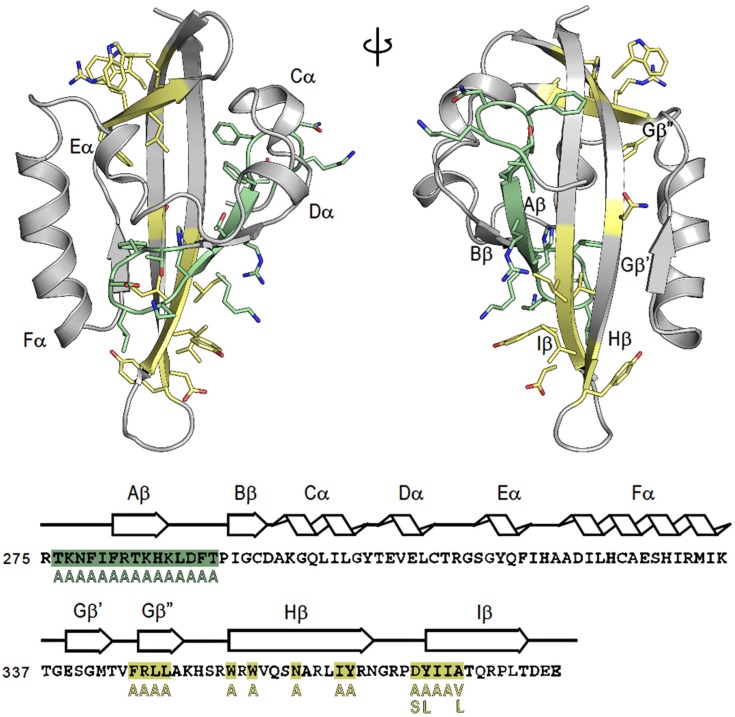
Structure and sequence of the mouse aryl hydrocarbon receptor (AhR) (Pubmed sequence: NP_038492.1) and map of generated mutations. The structure of the AhR PASB is taken from the homology model [33]. Structural elements of the AhR PASB homology model are indicated both in the structure and above the sequence. Mutants in A and B strands (N-terminal) are colored in green, while mutants in the G, H, and I strands (C-terminal) are colored in yellow.

**Figure 2:**
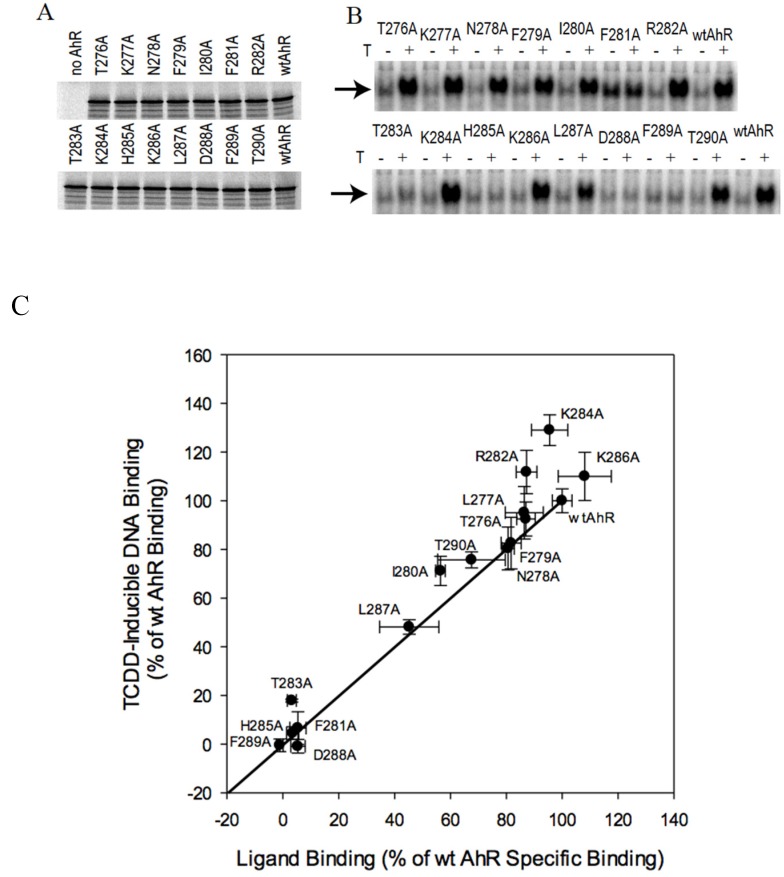
Effects of mutagenesis of amino acids 276–290 of the AhR PASB on 2,3,7,8-tetrachlorodibenzo-p-dioxin (TCDD)-inducible AhR transformation/DNA binding and [^3^H]TCDD specific binding. (**A**) In vitro protein expression of AhR mutants. Indicated AhR constructs were synthesized in vitro in the presence of [^35^S]-methionine and resolved by SDS-PAGE and autoradiography. (**B**) In vitro synthesized wild type (wt) or mutant AhRs and ARNT were diluted at 1:1:8 (*v/v/v*) ratio (AhR:ARNT:buffer), transformed in the presence of 10 nM TCDD (T) or 1% DMSO (*v/v*) for 3 h, and protein-DNA complexes analyzed by gel retardation analysis. A representative gel is shown and the specific protein-DNA complex is indicated with an arrow. (**C**) Comparison of ligand binding and ligand-dependent DNA binding for wt and mutant AhRs. [^3^H]TCDD binding to the in vitro synthesized AhR in the presence ARNT, diluted at 1:1:8 (*v/v/v*) ratio (AhR:ARNT:buffer), was analyzed by hydroxyapatite assay following 1 h of incubation at room temperature in the presence of 10 nM [^3^H]TCDD. Values were normalized to those obtained with unprogrammed lysate (non-specific binding control) and plotted vs corresponding values of the TCDD-inducible protein-DNA complex for each AhR from gel retardation experiments (panel A). Values represent the mean ± standard deviation of triplicate independent binding reactions. Data points overlapping with the trend line would indicate a proportional change in ligand binding and DNA binding for the indicated mutant AhR (i.e., unchanged transformation efficiency; see text). Note: F281A was constitutively active in DNA binding (2B); however, since it demonstrated no ligand-dependent increase in DNA binding, the resulting ligand-dependent change in DNA binding was low and similar to that of inactive AhRs (2B). Results presented are representative of at least three independent experiments.

**Figure 3:**
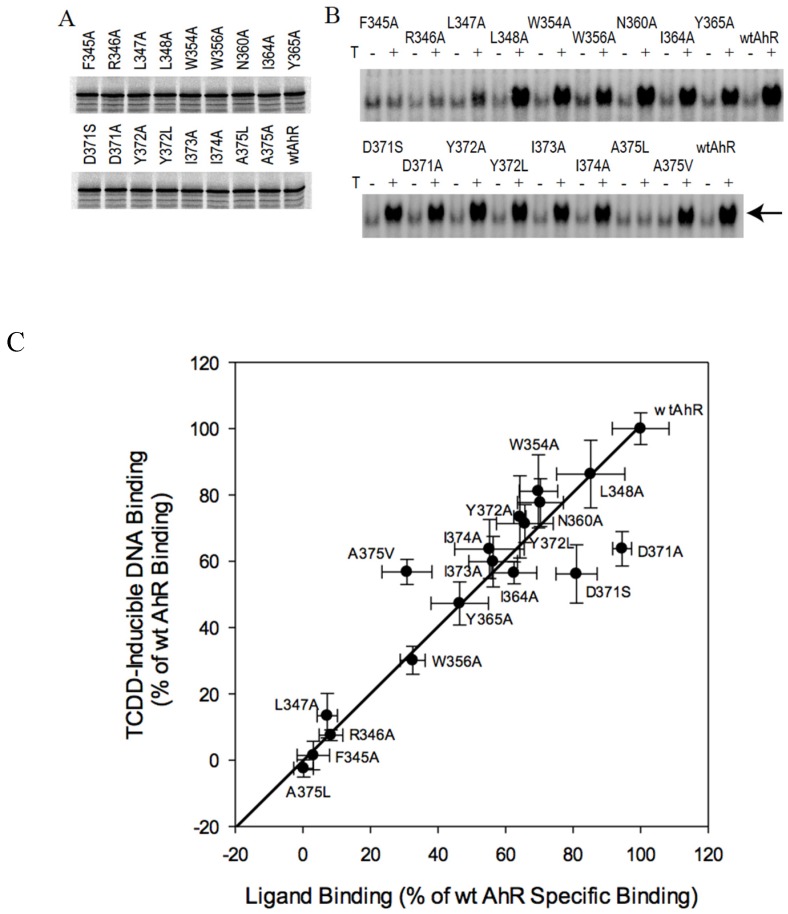
Effects of mutagenesis of select amino acids within residues 345–375 of the AhR PASB on TCDD-inducible AhR transformation/DNA binding and [^3^H]TCDD specific binding. (**A**,**B**). In vitro analysis of the wild type (wt) and mutant AhRs was as described in the legend to Figure 2. (**C**) Comparison of ligand binding and ligand-dependent DNA binding for wt and mutant AhRs was as described in the legend for Figure 2C. Note: mutations of Asp371 (D371A, D371S) demonstrate proportionally lower DNA-bound to [^3^H]TCDD-bound ratios, with experimental points to the right of the trend line. Values represent the mean ± standard deviation of triplicate independent binding reactions and the specific results presented are representative of at least three independent experiments.

Residues affecting hsp90 binding to the AhR PASB LBD have been previously identified and included Phe281, Thr283, His285, and several other residues in the A strand of the PASB β-sheet [23]. To analyze whether alterations in amino acids in this region would affect AhR transformation, alanine scanning mutagenesis of all residues within aa 276–290 (at the N-terminal end of the PASB domain) was carried out. Moreover, 14 residues located in the G, H, and I strands (at the C-terminal end of PASB) were also mutated in order to analyze the role of this region in the AhR transformation process. The locations of these residues within the protein structural elements are shown in Figure 1, both in the sequence and in the 3D representation of the homology model of the AhR PASB LBD [33]. Both groups of mutant AhRs were synthesized in vitro at levels similar to that of the wtAhR (Figure 2A and Figure 3A) and analyzed for their ability to bind [^3^H]TCDD and to transform and bind to DNA in a ligand-dependent manner. Ligand (TCDD)-dependent transformation and DNA binding was examined using gel retardation analysis.

### 2.2. N-Terminal Mutants: F281A Results in Constitutive Activation of AhR Transformation/DNA Binding

While ligand-dependent DNA binding was not affected by numerous mutations, most AhRs with mutations previously implicated in hsp90 binding [22] (T283A, H285A, D288A) and F289A failed to produce a ligand-dependent protein-DNA complex. In contrast, the F281A mutant AhR, also previously shown to be deficient in hsp90 binding [22], exhibited constitutively active (ligand-independent) transformation/DNA binding in gel retardation analysis (Figure 2B), although less DNA binding activity was observed relative to wtAhR.

All of the inactive AhR mutants and F281A failed to specifically bind [^3^H]TCDD (Figure 2C). The functional characteristics of the F281A mutant (decreased hsp90 binding, constitutive activation of DNA binding in vitro) are consistent with those of other constitutively active (ligand-independent) AhRs we have identified, including AhRdPASB and AhR/PASB-ARNT [21]. Constitutive transformation/DNA binding of hsp90 binding-deficient AhR mutants supports a role for the hsp90-binding site in maintaining the AhR in an inactive state in the absence of ligand. The remaining mutant AhRs demonstrated intermediate levels of ligand binding and transformation/DNA binding (Figure 2C), and, with a single exception (K284A), the transformation/DNA binding activity of these mutant AhRs was directly proportional to their relative ligand binding activity. These results are consistent with a direct positive relationship between ligand binding and DNA binding activities (Figure 2C), as well as with wtAhR at 100% ligand and DNA binding activity. Since the transformation efficiency is defined as the ratio of the quantity of DNA-bound to ligand-bound forms of the AhR, experimental points located at or close to the proportional trend line would possess similar levels of transformation efficiency to that of the wtAhR. Interestingly, the K284A mutant AhR demonstrated proportionally higher DNA binding than ligand binding and, therefore, a higher transformation efficiency than that of wtAhR (Figure 2C). This suggests that the K284A mutant AhR exhibits an altered transformation/DNA binding mechanism.

### 2.3. C-Terminal Mutants: D371A Impairs AhR Transformation

Among the C-terminal mutations (from F345 to A375), several (F345A, R346A, L347A, and A375L) exhibited little or no ligand-dependent DNA binding of the AhR (Figure 3B), similar to their lack of [^3^H]TCDD binding (Figure 3C). Interestingly, the A375V mutation impaired AhR ligand binding to a greater degree than DNA binding (compare ~30% to 55%, respectively). Previous analysis demonstrated that Ala375 is centrally located inside the ligand binding pocket and its mutation to Val results in a decrease in AhR ligand binding affinity that appears to be responsible for the lowered sensitivity of DBA mice (which contain V375A) to TCDD, compared to C57BL mice (which contain A375) [39,40]. For the majority of the remaining mutations, the relative ligand binding activity and ligand-dependent transformation/DNA binding activity of mutant AhRs were directly proportional (Figure 3C). Similar to the results in Figure 2C, the diagonal distribution of DNA binding versus ligand binding plot of the mutant AhRs (with some exceptions, as discussed below) indicates that, while their transformation efficiency was not significantly altered relative to that of the wtAhR, their overall functional activity did vary (Figure 3C). It appears that, although specific mutations within the PASB domain may result in decreased functional activity of the AhR protein, it does so without affecting its level of protein expression (Figure 3A). The mechanism(s) of this apparent decrease in functional response remains to be determined.

AhRs containing a mutation of Asp371 (D371A and D371S) varied significantly from the proportional trend line (Figure 3C), indicating proportionally lower DNA binding relative to ligand binding, i.e., they exhibited decreased transformation efficiency. This type of mutation would be consistent with an interference with the AhR transformation mechanism but not that of ligand binding. The difference in specific [^32^P]DRE-protein complex values between the wtAhR and D371A constructs was maintained in the presence of increasing [^32^P]DRE concentrations, indicating that this mutation did not reduce the apparent affinity of AhR DNA binding (data not shown). [^3^H]TCDD was bound to the wtAhR and D371A at similar levels and with a similar apparent affinity (K_d_ of 4.6 ± 0.3 nM and 5.0 ± 0.4 nM, respectively (mean ± standard deviation of three independent experiments)), indicating that this mutation did not affect TCDD binding to the AhR (data not shown). Together, these findings identify D371A as a novel residue affecting AhR transformation.

### 2.4. AhR Transformation Mutations in the PASA Domain

**Figure 4:**
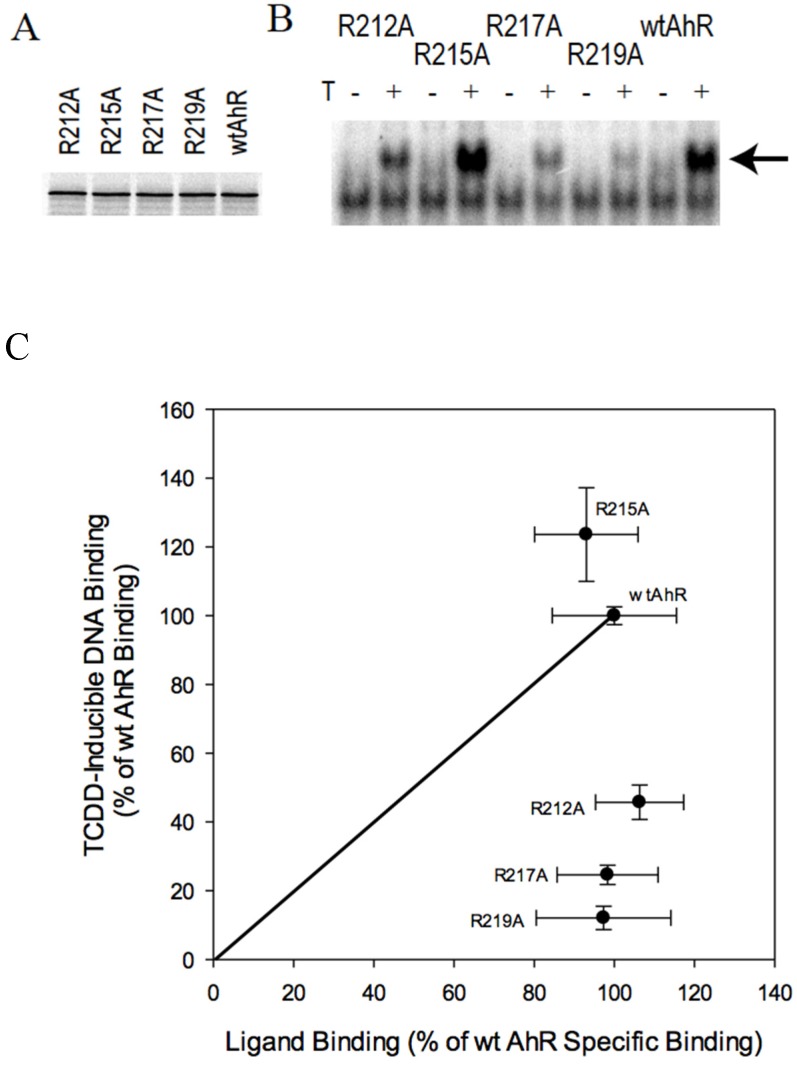
Effects of mutagenesis of select amino acids within the Arg-Cys-rich region on TCDD-inducible AhR transformation/DNA binding and [^3^H]TCDD specific binding. (**A**,**B**). In vitro analysis of the wild type (wt) and mutant AhRs was as described in the legend to Figure 2. (**C**) Comparison of ligand binding and ligand-dependent DNA binding for wt and mutant AhRs was as described in the legend for Figure 2C. Values are presented as the mean ± standard deviation of triplicate independent reactions, and the results presented are representative of at least three independent experiments.

In addition to the PASB domain, a highly conserved Arg-Cys-rich region located in the PASA domain of the AhR (aa 212–220) (Supplemental Figure S1) has been previously suggested to be important in transformation/DNA binding [41]. Within this region, we previously observed that Arg217 was likely involved in a ligand-dependent conformational change in the AhR, becoming more exposed following ligand binding, and this effect appeared to occur independently of the presence of ARNT [21]. Accordingly, mutations in the Arg-Cys-rich region could result in interference with ligand-dependent AhR transformation. To test this hypothesis, ligand-dependent transformation/DNA binding of AhRs containing mutations within the Arg-Cys-rich region (R212A, R215A, R217A, and R219A) were examined. All mutations were synthesized in vitro at levels similar to that of the wtAhR (Figure 4A). Of these four mutations, all but R215A resulted in decreased levels of TCDD-dependent AhR transformation/DNA binding (Figure 4B). The lack of effect of the R215A mutation is not surprising as this arginine is not present in the AhR of many species (Supplemental Figure S1). Since ligand binding was not reduced with any of these mutations (Figure 4C), the reduced DNA binding observed with the R212A, R217A, and R219A mutants resulted from impaired AhR transformation/DNA binding. While these results are similar to the effects observed with the D371A substitution, mutations in the Arg-Cys-rich region had a significantly greater impact on AhR transformation/DNA binding.

### 2.5. Effect of Sodium Molybdate on AhR Transformation

**Figure 5:**
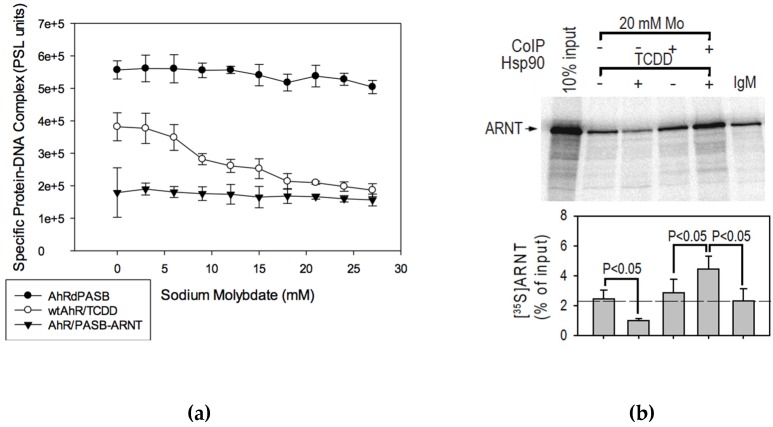
Effects of sodium molybdate on AhR transformation. (**a**) Transformation/DNA binding of the in vitro synthesized wtAhR is inhibited by sodium molybdate. The indicated in vitro synthesized AhR constructs were transformed in the presence of increasing concentrations of sodium molybdate for 3 h at room temperature and subjected to gel retardation analysis. For wtAhR, specific protein-DNA complex formation was determined as a difference in band intensities between TCDD (20 nM) and DMSO (1% (*v/v*)) reactions. Constitutively active AhRdPASB and AhR/PASB-ARNT were analyzed in the presence of DMSO (1% (*v/v*)). PSL = phospho-stimulated luminescence (imager output units). Values represent the mean ± standard deviation of three independent reactions, and the results are representative of three independent experiments. (**b**) Formation of a hsp90:AhR:ARNT ternary protein complex is stabilized by sodium molybdate. [^35^S]-labeled ARNT and unlabeled AhR were incubated in the presence of 20 nM TCDD (or DMSO (1% (*v/v*)) and 20 mM sodium molybdate (or control 20 mM sodium sulfate) for 1.5 h at room temperature prior to hsp90 co-immunoprecipitation. Specific [^35^S]-ARNT bands were quantitated and the values represent mean ± standard deviation of three independent reactions. Statistical analysis was performed using the Student’s *t*-test. Results are representative of two independent experiments.

Sodium molybdate has been previously found to inhibit AhR transformation/DNA binding, presumably due to stabilization of the ligand-free AhR complex with hsp90 and its co-chaperones [15,42]. Since many of the AhR mutants examined here and in previous studies [21,23] demonstrated decreased levels of hsp90 binding, incubation with sodium molybdate may reveal further functional differences among the wtAhR and these mutant AhRs. Accordingly, we first examined the effects of molybdate on AhR transformation/DNA binding by two previously identified [21] constitutively active AhRs, namely AhRdPASB (which lacks the PASB domain and does not bind hsp90) and AhR/PASB-ARNT (an AhR which contains the PASB domain of ARNT and still retains some hsp90 binding, presumably through interactions with the hsp90 binding site within the AhR bHLH domain). Gel retardation analysis revealed the concentration-dependent ability of molybdate to reduce TCDD-stimulated transformation/DNA binding activity of wtAhR by up to 70% (Figure 5a, Table 1). In contrast, molybdate had little effect on the transformation/DNA binding activity of the constitutively active AhRdPASB and AhR/PASB-ARNT proteins (Figure 5a, Table 1) over the range of sodium molybdate concentrations tested. Similarly, transformation/DNA binding of the constitutively active F281A mutant AhR was unaffected by the addition of 20 mM sodium molybdate (Table 1).

Except for AhRdPASB, which is known not to bind hsp90 in vitro or in cells in culture, the lack of effect of sodium molybdate on the remaining constitutively active AhRs was somewhat surprising given that both AhR/F281A and AhR/PASB-ARNT retain some hsp90 binding in vitro [21]. However, these results further confirm that the stabilization effect of molybdate on the AhR is mediated primarily by hsp90 interactions with the PASB LBD and little or no stabilization of hsp90 binding in the bHLH domain. Additionally, these effects are consistent with a mechanism of AhR transformation/DNA binding, in which sodium molybdate-sensitive ligand-dependent hsp90 dissociation in the PASB domain is a rate-limiting step in ligand-dependent initiation of AhR transformation. In such mechanism, the AhR mutants R217A and D371A would likely affect a step(s) distinct from that of the molybdate-sensitive initiation of AhR transformation/DNA binding, since molybdate-sensitive inhibition of transformation/DNA binding (Table 1) were similar between these mutant AhRs and that of wtAhR.

### 2.6. Molybdate Stabilization of An AhR:hsp90:ARNT Transitional Complex

While addition of sodium molybdate decreased wtAhR transformation/DNA binding (Figure 5a), its ligand binding was unaffected, resulting in an overall decrease in transformation efficiency of the wtAhR to approximately 30% relative to control (wtAhR in the presence of sodium sulfate). Sodium molybdate has been proposed to stabilize the AhR:hsp90 interaction [19,42], and could prevent effective displacement of hsp90 by ARNT from their overlapping binding sites in the PASB and bHLH domains. One intriguing possibility in this context would be the formation of a transitional ternary hsp90:AhR:ARNT complex that could be stabilized by molybdate. To examine this possibility, AhR and ARNT co-immunoprecipitation experiments were carried out with a hsp90 antibody in the absence or presence of sodium molybdate. In vitro synthesized wtAhR is readily co-immunoprecipitated by the anti-hsp90 antibody 3G3, whereas this antibody does not co-immunoprecipitate in vitro synthesized ARNT [21,24]. Therefore, co-immunoprecipitation of [^35^S]-labeled ARNT by the hsp90 3G3 antibody in a TCDD-inducible, AhR-dependent manner would reveal formation of the ternary hsp90:AhR:[^35^S]ARNT complex. In this experiment, AhR and [^35^S]ARNT-containing transformation reactions were incubated in the presence of TCDD (10 nM) or DMSO [1% (v/v)] and sodium molybdate (20 mM) or sodium sulfate (20 mM), followed by co-immunoprecipitation of hsp90 bound complexes with the 3G3 antibody, as previously described [21]. This co-immunoprecipitation experiment revealed a TCDD- and sodium molybdate-dependent increase in bound [^35^S]ARNT (Figure 5b), which was significantly greater than all relative controls. Additionally, no increase in [^35^S]ARNT was observed with molybdate in the absence of AhR (data not shown), demonstrating that the observed increase in [^35^S]ARNT co-immunoprecipitation was consistent with the proposed formation of a ligand-dependent ternary hsp90:AhR:[^35^S]ARNT complex [24]. The increase in [^35^S]ARNT signal was observed only in the presence of sodium molybdate, indicating the requirement of the stabilization of the TCDD:AhR:hsp90 interaction for detection of this transitional protein complex. In contrast, in the presence of ligand (TCDD) and absence of sodium molybdate, the hsp90 antibody co-immunoprecipitated significantly less [^35^S]ARNT than in the absence of ligand (to a level below that with the non-specific antibody IgM) (Figure 5b). This reduction likely reflects a decrease in available free [^35^S]ARNT, due to ligand-dependent AhR:[^35^S]ARNT dimerization, which would result in lower non-specific [^35^S]ARNT binding to the substrate. While the results of this co-immunoprecipitation complex are consistent with the formation of a molybdate-stabilized hsp90:AhR:ARNT complex, the exact geometry and stoichiometry of this complex remain to be determined. However, the observed formation of this transitional complex is consistent with the hypothesis of a multi-step mechanism of AhR transformation.

## 3. Discussion

Ligand binding to the AhR has been extensively studied, and the overall fingerprint amino acid residues (those presumably in contact with the bound ligand), as well as the amino acid residues implicated in differences in AhR affinity among many species, have been characterized [30,39,40,43,44]. Transcriptional activation by the ligand-transformed AhR:ARNT complex has also been a major focus of multiple studies resulting in the elucidation of the classical DRE-mediated pathway, as well as several alternative mechanisms (reviewed in [1,45,46]). However, the mechanistic events linking ligand binding and transcriptional activation (i.e., those constituting AhR transformation) remain poorly understood. Interest in this mechanism has been further increased by the observation that ligand-specific outcomes of AhR transformation and DNA binding (the classical DRE-dependent paradigm) cannot fully explain the diversity in AhR response [1,45,46,47,48].

**Figure 6:**
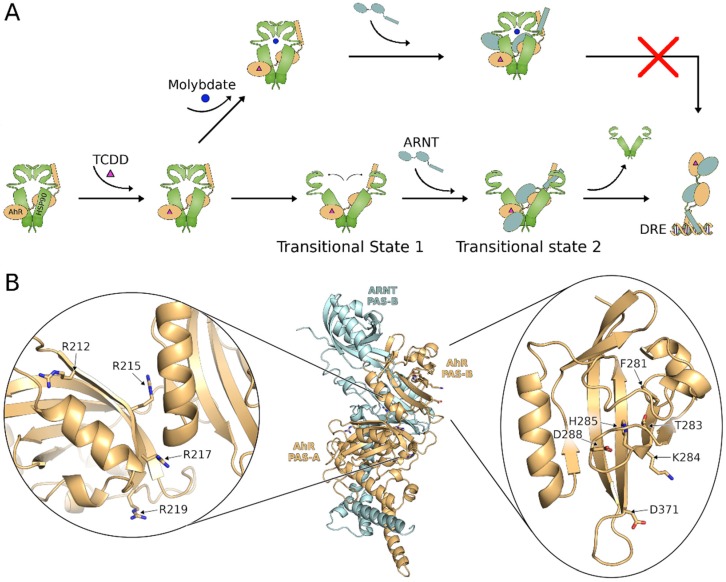
Multi-step mechanism of ligand-dependent AhR transformation involves interplay of hsp90 dissociation and dimerization with ARNT. (**A**) Proposed transitional mechanism of AhR transformation (details in text). (**B**). Homology model of the AhR:ARNT dimer [33] with close-ups of the AhR PASA Arg-Cys-rich region (left) and AhR PASB (right). Residues discussed in the text are represented with sticks and labeled.

Based on the results presented here and in previous studies [15,16,20,21,23,49,50,51], we propose a transitional states mechanism of AhR transformation (Figure 6A). In the figure, schematic structural representations are provided, based on some insights derived from other hsp90-protein complexes. In particular, while no structural information on the hsp90:AhR cytosolic complex is available to date, a recent Cryo-EM structure of hsp90 bound to another protein (CDK4) [52] provides interesting clues. It shows that hsp90 is in the closed conformation [53] and the client protein threads through the two hsp90 subunits due to a partial unfolding of two β-strands, leaving its two lobes on the opposite sides of the chaperone. By analogy, we hypothesize that the two AhR PAS domains could also arrange on the opposite sides of hsp90 (initial arrangement in Figure 6A), with the bHLH bound to the N-terminal domain of hsp90, as previously proposed [54]. Similarly to CDK4, the arrangement of the two PAS domains of AhR could be associated with a partial unfolding of the N-terminal β-sheets of the PASB. This proposal is supported by previous mutagenesis and Co-IP experiments showing that a set of residues at the N-terminal β-sheets of AhR PASB (F281, T283, H285, D288; see Figure 6B) are involved in both hsp90 association and ligand binding [23]. A possible explanation is that this region is partially unfolded when AhR is complexed with hsp90, with these residues exposed to participate in hsp90 interactions. A large PASB rearrangement occurring upon ligand binding could generate the folded state of the domain, predicted by our homology models [30,32,33], with the same residues pointing inside the cavity and being involved in ligand binding.

Initial ligand binding to the AhR has been suggested to proceed through a hypothetical reversible binding step but eventually results in formation of a non-reversible ligand-bound AhR complex [15,49]. It is likely, although not yet experimentally demonstrated, that the reported irreversible binding of ligand to the AhR correlates with ligand-dependent displacement of hsp90 from its binding site within the PASB domain ligand binding site [15,24,49,55]. According to our structural hypothesis, the large conformational change in the PASB domain of AhR occurring upon ligand binding could trigger the switch of hsp90 to the open form (*transitional state 1* in Figure 6A). In this state, the NLS motif of AhR is exposed and the AhR:hsp90 complex translocates into the nucleus. Previous evidence that the Arg-Cys-rich region (Figure 6B) in AhR PASA becomes more exposed following ligand binding [21] supports the hypothesis that the uncovering of this part of the AhR PASA domain could be due to an opening hsp90 and/or to a change in AhR conformation, thus facilitating the dimerization with ARNT. The alteration of the hydrophobicity of this region with mutation of Arg to Ala (Figure 4) could result in an altered stability of the open form, affecting the subsequent steps of the mechanism.

Once the AhR:hsp90 complex enters the nucleus, ARNT binds to the complex forming the *transitional state 2* (Figure 6A), and this is immediately followed by the progressive release of hsp90 and the formation of the transcriptionally active state. The existence of this transitional state is supported by the molybdate experiments (Figure 5). Molybdate is known to stabilize a closed conformation of hsp90 [56]; thus, it is reasonable that opening of hsp90 and AhR displacement are inhibited by molybdate addition (Figure 6A). Our results indicate that, while sodium molybdate decreases AhR transformation efficiency as a result of stabilization of AhR:hsp90 binding [19,42], it does not inhibit the binding by ARNT but does inhibit ARNT-mediated displacement of hsp90. Therefore, the ternary hsp90:AhR:ARNT complex (Figure 5b) would represent *transitional state 2* in this mechanism (Figure 6A). The lack of sodium molybdate effects on the constitutively active AhR mutants, which either lack or have an altered hsp90:PASB binding [21,23], is consistent with the idea that it is hsp90 dissociation from the PASB binding site that is blocked by molybdate. Hsp90 dissociation from its bHLH binding site is not blocked by molybdate and the dimerization of ARNT, with the AhR results in displacement/disruption of hsp90 binding within the bHLH domain and formation of the fully competent DNA binding form of the AhR:ARNT complex.

Two other mutations in the PASB highlighted in this work may be interpreted on the basis of the proposed mechanism (Figure 6B). F281A is the only single-point mutation that resulted in constitutive activation of AhR. Interestingly, F281 is the N-terminal residue of the PASB A-strand (that we hypothesized to partially unfold to allow the AhR association with hsp90), and it has a key role in ligand binding. Thus, F281 could be the position responsible for the initiation of the transformation mechanism. The decreased transformation/DNA binding efficiency observed with the D371A AhR mutant in this study suggests that this mutation may alter the ligand dependent dissociation of AhR from hsp90 (*transitional state 1*) and, therefore, would likely interfere with a downstream step(s) in the transformation mechanism. D371 is indeed located at the beginning of the I-strand, near to the HI loop (Figure 1), a position far from the interface with ARNT [33]. However, given the structural similarity of the PAS fold with the N-lobe of CDK4 (data not shown), this loop could be at the interface with hsp90. Since hsp90 binding levels of the D371A mutation were similar to that of wtAhR (Supplemental Figure S2), the reduction in its efficiency of AhR transformation could result from an alteration in ligand-dependent dissociation of AhR from hsp90 in presence of ARNT.

Our homology model of the AhR:ARNT heterodimer (Figure 6B) and previous mutagenesis experiments [32,33] identified several mutations in the AhR PASA domain and only one within the PASB, I324R, that negatively affects AhR dimerization with little effect on [^3^H]TCDD ligand binding [32]. The K284A mutation, developed in this work, demonstrated a somewhat disproportionate transformation ratio, i.e., DNA-bound to ligand-bound AhR (Figure 2C), that would be consistent with an effect on AhR transformation. Despite the fact that K284 lies in the A strand (Figure 6B), it was shown that this residue is not involved either in ligand binding nor in hsp90 association [23]. Recently, a computational study of the homologous HIF-2α:ARNT dimer [57] showed that the ARNT PASA FG loop strongly interacts with the PASB of HIF-2α in the region, corresponding to the A-strand of AhR (in the folded state), which includes K284. On this basis, we propose that similar interactions are present in the AhR:ARNT dimer; thus, the observed K284A higher transformation efficiency could be explained by an improved AhR:ARNT interaction.

The proposed mechanism implies a somewhat complex sequence of events for hsp90 dissociation. Not only does hsp90 dissociation take place in a site-restricted manner (separately, from the PASB and bHLH sites), but, within the PASB site, it appears to occur in two distinct steps, ligand-dependent displacement of hsp90 associated with the rearrangement of the ligand binding domain (leading to transitional state 1), followed by ARNT binding (leading to transitional state 2) that promotes complete hsp90 displacement (Figure 6A). The overlap between the hsp90 binding site in the PASB domain and the ligand binding site (amino acid residues F281, T283, H285, and D288 in Figure 6B), as well as the observation that TCDD, induces hsp90 dissociation from AhR PASB fragments that lack the bHLH hsp90 binding site [21,22] suggest that ligand binding alters the structure of the AhR PASB LBD and its interactions with hsp90. By analogy with the CDK4 system [52], the AhR PASB could undergo a conformational change in its N-terminal region (A-strand) that, upon ligand binding, displaces residues F281, T283, H285, and D288 from the hsp90 interaction interface and directs their side-chains toward the interior of the cavity.

While the PASA domain by itself does not bind hsp90 nor does it significantly stabilize hsp90 binding to the adjacent PASB domain, its role in hsp90 dissociation from a PASA-PASB fragment is likely through its ability to initiate dimerization with ARNT [16,21,24]. The Arg-Cys-rich region is located at the C-terminus of the AhR PASA domain, and it becomes more exposed following ligand binding. Presumably, the ligand-dependent displacement of hsp90 from PASB also exposes that region of the AhR PASA domain [21], as a result of the hsp90/AhR conformational change leading to the transitional state 1. Then, the exposed PASA can recruit ARNT and form the ternary hsp90:AhR:ARNT complex (transitional state 2), thus facilitating the complete release of hsp90 and formation of the AhR:ARNT high affinity DNA-binding form.

The AhR PAS domains appear to possess the necessary functional features to be inactive in the absence of ligand, as well as activated in the presence of ligands, independently of the bHLH domain. Deletion of the AhR PASB domain eliminated hsp90 binding to the AhR, removed the inhibitory function of the PASB on AhR transformation efficiency, and resulted in a constitutively active AhR [16,21,51,58]. Ligand-dependent displacement of hsp90 from the PASB could be an initial and sufficient structural change that triggers AhR transformation through changes in conformation and interactions of the AhR:hsp90 complex and/or within AhR domains [16,21,24,43]. In this mechanism, the ability of a ligand to displace or modify hsp90 binding to the PAS domains, rather than the general ability of ligand to simply bind within the PASB, would be predictive of its AhR activation potential.

## 4. Materials and Methods

### 4.1. Chemicals and Antibodies

TCDD was obtained from Dr. Stephen Safe (Texas A&M University, College Station, TX, USA) and [^3^H]TCDD (13 Ci/mmole) from ChemSyn Laboratories (Lenexa, KS, USA). Sodium molybdate dihydrate was from Mallinckrodt (St. Louis, MO, USA). All other chemicals were of analytical grade or better and obtained from Fisher Scientific (Waltham, MA, USA. or Sigma-Aldrich (St. Louis, MO, USA). The monoclonal 3G3p90 anti-hsp90 antibody [24] was produced at Antibodies Incorporated (Davis, CA, USA) from hydridoma cells kindly provided by Dr. Gary Perdew (Penn State University, University Park, PA, USA). All other antibodies were from Santa Cruz Biotechnology (Santa Cruz, CA, USA).

### 4.2. Plasmid Constructs

mβAhR/pcDNA3, mβAhRdPASB/pcDNA3, mβAhR/PASB-ARNT/pcDNA3, and mβArnt/pcDNA3 have been previously described [21,59]. Point mutations of mβAhR/pcDNA3 were carried out using the QuikChange technique (Agilent, Santa Clara, CA, USA) and all constructs all constructs were verified by sequencing.

### 4.3. In vitro Expression

Wild-type (wt) and mutant AhRs were synthesized in vitro in the presence of [^35^S]-L-methionine (Perkin Elmer, San Jose, CA, USA) or unlabeled L-methionine using the TNT Quick coupled transcription/translation rabbit reticulocyte lysate kit (Promega, Madison, WI, USA). Aliquots of the in vitro synthesized proteins were analyzed with SDS-PAGE as previously described [21].

### 4.4. Hydroxyapatite (HAP) Ligand Binding Assays

[^3^H]TCDD specific binding to the in vitro synthesized proteins diluted in MEDG (25 mM MOPS (morpholinepropanesulfonic acid; pH 7.5), 1 mM EDTA, 1 mM dithiothreitol (DTT), 10% [vol/vol] glycerol) buffer containing 150 mM KCl (conditions used for AhR in gel retardation analysis) was conducted as previously described in detail [21,60]. Binding reactions were also carried out in the presence of 20 nM [^3^H]TCDD and equivalent amounts of unprogrammed in vitro synthesized reactions were used as the non-specific binding control. For affinity measurements, in vitro reactions and non-specific binding controls were incubated in the presence of increasing concentrations (1-20 nM) of [^3^H]TCDD for 1 h at room temperature, and relative affinity values were determined using non-linear regression plots (SigmaPlot (Systat Software), San Jose, CA, USA).

### 4.5. Gel Retardation Assays

Wt and mutant AhRs and ARNT were synthesized in vitro in the presence of unlabeled L-methionine using the TNT Quick coupled transcription/translation rabbit reticulocyte lysate kit (Promega). The resulting AhR and ARNT translation reactions were mixed with MEDG containing 150 mM KCl (for wt and mutant AhRs) or MEDG without added KCL (for AhRdPASB or AhR/PASB-ARNT) in a 1:1:8 (*v/v/v*) ratio and incubated with the indicated concentration of TCDD or 1% (*v/v*) DMSO (the solvent control) for the indicated periods of time at room temperature. Double-stranded oligonucleotides containing the AhR:ARNT DNA binding site (DRE3) from the murine *CYP1A1* upstream regulatory sequence were ^32^P-labeled, and gel retardation analysis was conducted with the transformed AhR reactions as detailed previously [60,61]. For saturation binding analysis, incubation reactions contained increasing amounts of the ^32^P-labeled DRE (0.3–1.7 µmol). Gels were visualized using an FLA9000 Fujifilm Imager (Walnut Creek, CA, USA) and quantitated with Fujifilm Multi Gauge software.

### 4.6. Co-Immunoprecipitation Assay

Hsp90 co-immunoprecipitation analysis of the in vitro synthesized proteins using the 3G3 anti-hsp90 antibody was as previously described [21]. Where indicated, 20 mM sodium molybdate was included in transformation reactions and the co-immunoprecipitation washing buffer. For transitional complex analysis, a 1:1 mixture of in vitro synthesized [^35^S]ARNT and AhR was incubated in the presence of 20 nM TCDD (or 1% v/v solvent control DMSO) and/or 20 mM sodium molybdate (or 20 mM of control sodium sulfate) for 1.5 h at room temperature prior to co-immunoprecipitation. Maintenance of COS-1 cells, transient transfections and consecutive hsp90 co-immunoprecipitation analysis from COS-1 cell lysates were previously described [23].

### 4.7. Statistical Analysis

Analysis of the statistical significance of differences of experimental values was conducted using the Student’s *t*-test in Excel (Microsoft). Determination of ligand or DNA binding affinity was conducted using regression analysis of the saturation binding curves in SigmaPlot.

### 4.8. AhR PASB LBD Homology Model

The AhR homology model, previously developed [33] on the basis of the HIF-2α:ARNT template [62], was used to generate the three-dimensional representations using PyMOL Version 1.6 [63].

## Figures and Tables

**Table 1:** Effects of sodium molybdate on AhR transformation/DNA binding in vitro.

AhR Mutation	Mode of Activation	Percent Inhibition by Molybdate
AhRdPASB	Constitutively Active	4.3 ± 5.8 ^a^
AhR/PASB-ARNT	Constitutively Active	9.1 ± 4.1
AhR F281A	Constitutively Active	5.0 ± 12.1
AhR R217A	Ligand-Dependent	67.1 ± 7.3 ^b^
AhR D371A	Ligand-Dependent	61.7 ± 11.5 ^b^
wtAhR	Ligand Dependent	69.6 ± 6.6 ^b^

^a^ Values represent the mean ± SD of at least triplicate independent determinations. ^b^ Statistically significant inhibition compared to the absence molybdate at *p* < 0.05 as determined by the Student’s *t*-test.

## Supplementary Materials

Supplementary materials can be found at https://www.mdpi.com/1422-0067/21/7/2474/s1.
