# Supplementary material for: Transitional States in Ligand-Dependent Transformation of the Aryl Hydrocarbon Receptor into Its DNA-Binding Form
Source: Int J Mol Sci. 2020 Apr 2;21(7):2474. doi: 10.3390/ijms21072474 (PMC7177239; doi:10.3390/ijms21072474)

Supplemental Figure S1. Sequence alignment of the Arg-Cys region
of AhRs from diverse species. Conserved Arg residues are indicated
in blue and Cys residues in yellow.

Species	Protein	Sequence
		* **
Mouse (C57)	AhR[b]	NASFMERCFRCRLRCLLDNS
Guinea Pig	AhR	NSPLMERCFVCRLRCLLDNS
Rat (RN)	AhR	NTAFMERCFRCRLRCLLDNS
Human	AhR	NSPLMERCFICRLRCLLDNS
Hamster	AhR	SASFLERCFICRLRCLLDNS
Rabbit	AhR	NSSFMERCFICRLRCLLDNS
Seal	AhR	NSSCMERSFVCRLRCLLDNS
Whale	AhR	NSSSMERCFVCRLRCLLDNS
Dolphin	AhR	NSSSMERCFVCRLRCLLDNS
Chicken	AhR	NSSFMERNFICRLRCLLDNS
Tern	AhR	NSSFMERNFICRLRCLLDNS
Trout	AhR	NSSFLERNFVCRFRCLLDNS
Fundulus	AhR1	NSSFLERNFVCRFRCLLDNS
Fundulus	AhR2	SSSFLERSFVCRFRCLLDNS
Zebrafish	AhR1a	NSTCLERNFICRLRCLLDST
Zebrafish	AhR1b	NSSFLERNFVCRFRCLLDNS
Zebrafish	AhR2	NSSFLERSFCCRFRCLLDNS
Tomcod	AhR	NSSFLERSFVCRFRCLLDNS
Lamprey	AhR	NSSFLERQFICRFRCLLDNS

Supplemental Figure S2.

Hsp90 binding to an AhR containing a D371A mutation is similar to that of wild-type (wt) AhR. D371A and wtAhR are indicated with an asterisk. Hsp90 binding to other AhR mutants are also shown, but are not included in this manuscript.

COS-1 cells grown in 100 mm culture plates were transiently transfected with 8 μg of wtAhR or the indicated mutant AhR expression vector and 20 μl of Lipofectamine 2000 (Invitrogen). Twenty four hours after transfection, cells were rinsed with PBS, plates were scraped and cells lysed on ice for 30 min using MEG-RIPA buffer (25 mM MOPS pH 7.5, 10% v/v glycerol, 1 mM EDTA, 1% v/v Igepal CA-630, 0.5% w/v sodium deoxycholate, 0.1% w/v SDS, 0.5% v/v protein inhibitor cocktail (Sigma)). Lysates were centrifuged at 14,000xg for 10 min, and 400 μl aliquots of supernatant (0.8-1.2 mg protein) were incubated with anti-hsp90 antibody 3G3 or control IgM bound to Affigel 10 substrate (BioRad) (prepared as previously described (21,23)) for 1 h at 4°C with shaking. Samples were washed three times with MEGRIPA. Proteins were resolved by SDS-PAGE and detected by Western blotting as described (20) using anti-AhR M20 antibody (Santa Cruz Biotechnology), anti-hsp90 antibody 3G3 and anti-β-actin antibody (Santa Cruz Biotechnology). Results are representative of three independent experiments.

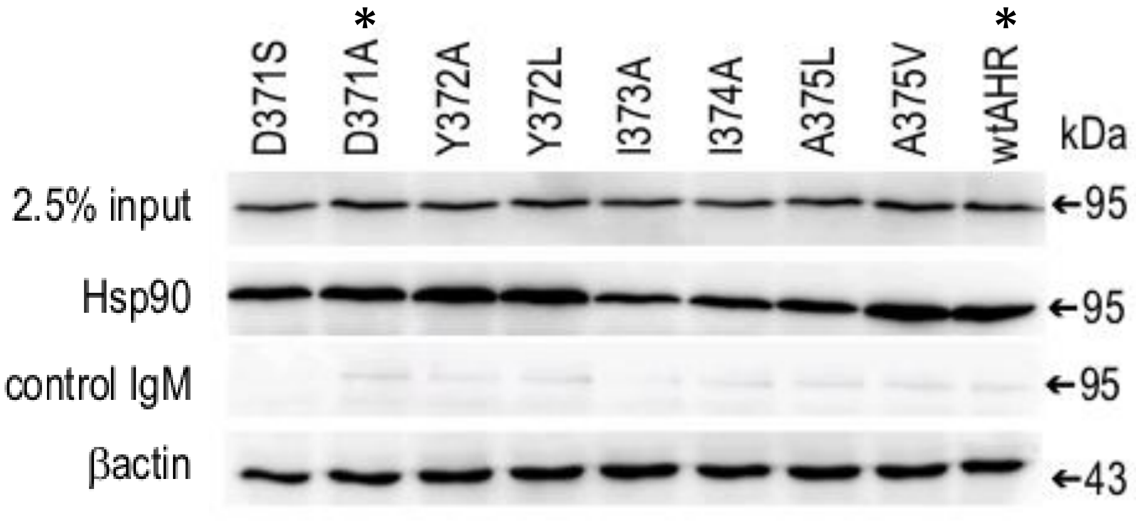

Supplement: Supplementary file 1 [file ijms-21-02474-s001.pdf]
